# How the Unfolded Protein Response Is a Boon for Tumors and a Bane for the Immune System

**DOI:** 10.4049/immunohorizons.2200064

**Published:** 2023-04-17

**Authors:** Lydia N. Raines, Stanley Ching-Cheng Huang

**Affiliations:** * Department of Pathology, Case Western Reserve University School of Medicine, Cleveland, OH; † Case Comprehensive Cancer Center, Case Western Reserve University School of Medicine, Cleveland, OH

## Abstract

The correct folding of proteins is essential for appropriate cell function and is tightly regulated within the endoplasmic reticulum (ER). Environmental challenges and cellular conditions disrupt ER homeostasis and induce ER stress, which adversely affect protein folding and activate the unfolded protein response (UPR). It is now becoming recognized that cancer cells can overcome survival challenges posed within the tumor microenvironment by activating the UPR. Furthermore, the UPR has also been found to impose detrimental effects on immune cells by inducing immunoinhibitory activity in both tumor-infiltrating innate and adaptive immune cells. This suggests that these signaling axes may be important therapeutic targets, resulting in multifaceted approaches to eradicating tumor cells. In this mini-review, we discuss the role of the UPR in driving tumor progression and modulating the immune system’s ability to target cancer cells. Additionally, we highlight some of the key unanswered questions that may steer future UPR research.

## Introduction

The endoplasmic reticulum (ER) is an important organelle whose proximity to other organelles, such as the nucleus, mitochondria, and Golgi apparatus, exemplifies its position as a key regulator of global cellular processes (1). The most classical role of the ER is protein translation, where approximately one-third of all cellular proteins are made (2, 3). During this process, proteins are carried into the ER lumen by chaperones that mediate protein folding through a series of complex modifications such as *N*-linked glycosylation, disulfide bond formation, and proline isomerization. Proper protein folding is essential for cell survival, and this process is subject to high levels of quality control, where the disruption of any of these processes can induce a phenomenon known as ER stress.

The most classical inducer of ER stress is the accumulation of unfolded or misfolded proteins that can result in the unfolded protein response (UPR), a process through which integrated signaling pathways activate to re-establish homeostasis. Moreover, recent evidence has shown that environmental factors such as hypoxia, nutrient deprivation, acidosis, and exogenous lipids are also able to interrupt ER proteostasis and trigger the UPR (4, 5). UPR activation serves to maintain ER homeostasis via a series of intricate mechanisms, including the transcriptional regulation of protein folding machinery, the degradation of cytosolic mRNAs, the inhibition of protein translation inhibition, and the breakdown of misfolded proteins using the ER-associated protein degradation (ERAD) system. Whereas UPR induction can be productive in resolving ER stress and restoring homeostasis, unproductive or chronic UPR activation can lead to apoptosis (6, 7). Importantly, UPR signaling has been associated with transcriptional or metabolic regulation linking cellular function, host defense, cytokine production, Ag presentation, and immunological tolerance (5). The presence of stresses in the tumor microenvironment (TME) prompts the question of whether the activation of UPR benefits cancer cell tumorigenesis and whether these stress responses pose positive or detrimental effects on the immune system. Therefore, it is crucial to understand the multifaceted role of UPR signaling and its potential implications in the context of cancer and the immune system.

The mechanisms that dictate the activation of the UPR and the actions of the individual arms of the UPR have been described extensively elsewhere (1, 5, 8, 9). In this mini-review, we focus primarily on how the arms of the UPR work together or separately to induce various aspects of cancer cell tumorigenesis or influence immune cell effector function in the TME.

### The UPR

In multicellular organisms, activation of the UPR involves the coordinated action of three ER transmembrane sensors: inositol-requiring enzyme 1α (IRE1α), protein kinase RNA-like ER kinase (PERK), and activating transcription factor 6 (ATF6) (1). Under homeostatic conditions, these three sensors lie inert through an association with the chaperone BiP (encoded by *GRP78*) (10); however, under stress conditions, BiP will dissociate from these sensors, allowing for their activation (7, 11). BiP dissociation is attributed to the fact that it has a higher affinity for unfolded or misfolded proteins than it does for the sensors themselves (7, 11), and once BiP is dissociated, the individual sensors are able to oligomerize and autophosphorylate, resulting in the activation of a basic leucine zipper (bZIP) family transcription factor (5).

#### IRE1α

The dissociation of BiP from IRE1α and subsequent autophosphorylation induces a conformational change that reveals a C-terminal domain with endonuclease activity (7). This C-terminal endonuclease mediates the nonconventional splicing of the mRNA X-box binding protein 1 (XBP1) in the cytosol (12, 13). Splicing of XBP1 (XBP1s) allows it to translocate to the nucleus where it acts as a transcription factor to promote the transcription of ER chaperones and protein-folding enzymes (14, 15). These genes include components that facilitate the degradation of cytosolic RNA via ERAD. In this process, misfolded proteins are transported to the cytosol and subsequently broken down by the 26S proteasome, providing the defensive benefits of UPR activation (8, 16). Furthermore, XBP1s targets have recently expanded and are now known to include genes involved in lipid metabolism (17–19), proinflammatory cytokine production (20–22), HIF1α-mediated hypoxic responses (23), cellular differentiation (24), and hexosamine biosynthesis (25). Aside from XBP1 splicing, prolonged IRE1α activation can trigger an XBP1-independent pathway referred to as regulated IRE1α-dependent decay (RIDD) (26, 27). This pathway enables the degradation of mRNAs within the cytosol, limiting protein translation. Nevertheless, the induction of RIDD during severe or chronic ER stress has been associated with apoptosis (26, 28).

#### PERK

Similar to IRE1α, PERK is activated via homodimerization and autophosphorylation, which activate a C-terminal kinase domain (10); however, instead of unconventional splicing of an mRNA, PERK activation phosphorylates eIF2α, a protein that suppresses global protein translation by interfering with 5′-cap assembly (29–31). This attenuation of protein synthesis is necessary so that proper care can be taken to degrade existing misfolded proteins, but not all protein synthesis is attenuated. Loss of 5′-cap assembly instead enhances cap-independent protein translation including that of the transcription factor ATF4 (32). ATF4 is involved in mediating numerous key processes within the cell such as amino acid metabolism (33, 34), redox homeostasis (35, 36), and autophagy (37, 38). In situations where ER stress cannot be resolved, PERK-ATF4 signaling induces cell apoptosis through the transcriptional regulation of DNA damage inducible transcript 3 (DDIT3; also known as CHOP). CHOP is initially repressed during the early phase of the UPR but increases as ER stress continues unresolved (39, 40). If ER stress is able to be resolved, CHOP and ATF4 can instead complex together to reinstate protein translation by inducing the expression of GADD34 to dephosphorylate eIF2α, allowing mRNA translation to begin anew (41).

#### ATF6

ATF6 is the third member of the UPR and an ER-resident transmembrane protein (5). Yet, unlike PERK or IRE1α, ATF6 itself translocates to the Golgi apparatus, where it undergoes proteolysis by site 1 protease (S1P) and S2P to liberate an active soluble N-terminal fraction called ATF6p50. ATF6p50 migrates to the nucleus as a transcription factor to induce the expression of genes related to ER capacity and folding as well as the ERAD pathway (12, 42). Interestingly, many of ATF6p50’s target genes overlap with XBP1s targets, suggesting some redundancy between the two pathways (43).

### The UPR in tumor cells

**FIGURE 1.**
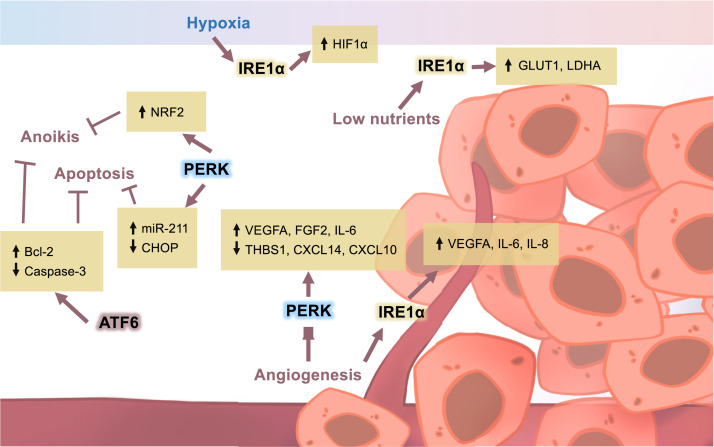
Activation of the UPR in cancer cells promotes cell survival and progression. Under hypoxic conditions, tumor cells can induce the expression of IRE1α, which complexes with HIF1α to promote resistance to hypoxia. Tumor cells can induce PERK to suppress caspase-mediated apoptosis. IRE1α can also induce the expression of GLUT1 and LDHA, allowing tumor cells to sequester more glucose and enhance glycolysis. In low-nutrient conditions, angiogenesis can be modulated by the signaling of PERK and IRE1α. To prevent cell death and enable metastasis, PERK and ATF6 can independently inhibit apoptotic mechanisms through enhanced miR-211 and Blc-2, respectively. Furthermore, PERK and ATF6 may protect against anoikis through NRF2 and Bcl-2, allowing tumor cells to migrate to new tissues.

As mentioned previously, many of the key environmental factors found in the tumor niche are capable of provoking ER stress to activate the UPR, and for tumor cells, this presents a significant hurdle that must be overcome to survive. Because of this immense survival pressure, the induction of the UPR is starting to become recognized as a crucial means through which tumor cells overcome these environmental pressures while at the same time evading immune challenges (Fig. 1). This can be further highlighted by the fact that increased activity of ER stress signaling in tumors has corresponded with worse patient prognosis and adverse treatment outcomes in numerous cancers such as melanoma, osteosarcoma, and breast and bladder cancer (44–48).

As tumor cells become established and begin to expand, they undergo the Warburg effect, a form of aerobic glycolysis that allows for rapid energy generation at the cost of increased lactate production (49, 50). Lactate is then released by the tumor cells into the surrounding microenvironment causing a decrease in pH. At the same time, increases in nucleotide synthesis promote proliferation, which requires tumor cells to consume more oxygen, thus increasing hypoxia. Importantly, because protein folding requires oxygen, the increasingly hypoxic environment imposes stress on the protein folding and synthesis demands in tumor cells (51). Evidence from triple-negative breast cancer shows that IRE1α signaling becomes induced, generating XBP1s. XBP1s is then able to complex with other transcriptional regulators such as HIF1α to promote resistance to hypoxia (23, 52). Levels of extreme hypoxia (<0.02% oxygen) were also found to induce PERK/ATF4 signaling in transformed mouse embryonic fibroblasts in vitro and in vivo (53). Loss of PERK in these cells was associated with increased expression of apoptotic markers caspase-12, caspase-3, and poly(ADP-ribose) polymerase (PARP), suggesting that PERK may also mediate cell survival during hypoxia by suppressing caspase-mediated cell death (53). Taken together, these findings demonstrate that IRE1α and PERK aid the tumor cells against hypoxia by upregulating resistance genes and suppressing apoptosis.

In addition to hypoxia resistance, induction of IRE1α-XBP1 was found to regulate the expression of key targets of Hif1α, including glucose transporter 1 (GLUT1) and lactate dehydrogenase A (LDHA) (23). GLUT1 is critical for the uptake of glucose into the cell, which is a key fuel source for glycolysis. Additionally, LDHA plays a crucial role in the breakdown of pyruvate into lactate, which can then be released into the extracellular environment.

Then, as the tumor continues to grow, compounding nutrient deprivation requires the cancer cells to divert circulating nutrients to the TME through the process of angiogenesis. In glioma, it has been suggested that the loss of IRE1α in tumor cells was correlated with decreased angiogenesis via the reduction of proangiogenic factors such as VEGFA, IL-6, and IL-8 (54). PERK was also found to mediate angiogenesis in both human and mouse cancer cells where the loss of PERK resulted in malformed vessels. This effect was found to be the result of PERK-ATF4–mediated transcriptional regulation of proangiogenic factors such as VEGFA, FGF2, and IL-6 and suppression of antiangiogenic factors such as thrombospondin-1 (THBS1), CXCL14, and CXCL10 (55, 56). Importantly, ATF4 was demonstrated to bind directly to the regulatory site of VEGF. These findings suggest that PERK and IRE1α could potentially act cooperatively to ensure angiogenesis.

In addition to nutrient acquisition, the UPR is thought to protect the growing tumor cells from apoptosis. In cervical cancer cells, increased ATF6 was associated with protection against apoptosis by increasing Bcl-2 expression via MAPK signaling (57). Moreover, when ATF6 was silenced in these cells, Bcl-2 expression decreased whereas caspase-3 expression increased, suggesting a role for ATF6 in mediating protection against cell death (57). A similar effect was also observed in p53-mutant triple-negative breast cancer (58).

Although ATF6 was presumed to mediate antiapoptotic factors in some cancers, a role for PERK has also been suggested. One example is miR-211, a prosurvival microRNA whose induction is dependent on the PERK signaling cascade. When miR-211 is induced, it can directly bind to the CHOP promoter, which increases histone methylation at H3K27 and transcriptional repression of the site (40). This PERK/miR-211 axis has been observed in several murine mammary tumors and primary human B cell lymphomas (40). Recently, it was discovered that PERK may also serve as a means of protection against paraptotic cell death in melanoma cells. In this study, the loss of PERK induced paraptosis of melanoma cells, releasing tumor Ags that then promoted type I IFN production in tumor-associated dendritic cells (DCs), resulting in increased antitumor immunity (59). How PERK regulates this type of cell death is not completely understood and could potentially be linked to the loss of miR-211.

Finally, once the tumor cell has sufficiently expanded, established new vasculature, and suppressed internal cell death mechanisms, it will eventually undergo metastasis and migrate to distal tissue sites. To facilitate this process, PERK has been speculated to protect against anoikis, a type of cell death that is induced when cells lose contact with neighboring cells and the extracellular matrix (60–63). As metastatic cells enter the circulation, the harsh environment and oxidative stress stimulate PERK signaling, which then activates an antioxidant mechanism through NRF2 (35, 63, 64). It is also noteworthy that the anoikis resistance mechanism includes increased expression of Bcl-2, which, as mentioned above, may be mediated by ATF6 signaling. This may suggest that ATF6 and PERK act in concert to suppress numerous types of cell death to ensure tumor progression and metastasis.

Recent evidence regarding the activation of the UPR seems to overwhelmingly conclude that these signaling pathways play beneficial roles in tumorigenesis. However, these beneficial roles do not stop at intracellular mechanisms. In the following section, we provide an overview of how induction of ER stress and the activation of UPR in immune cells can exhibit dire consequences against antitumor immunity.

### The UPR in tumor-infiltrating immune cells

**FIGURE 2.**
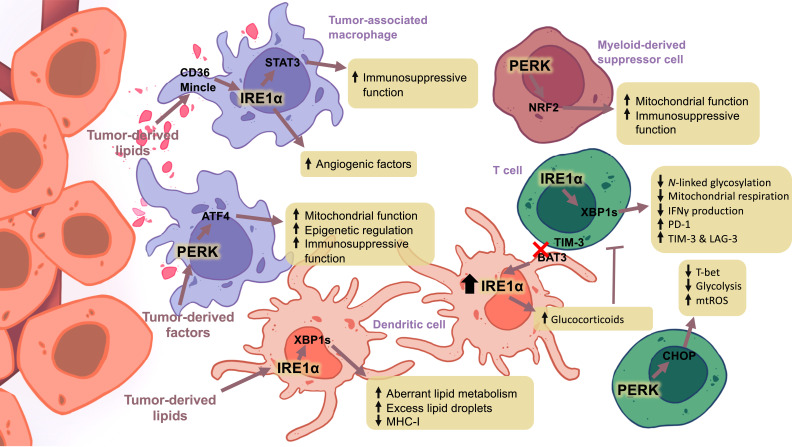
Induction of the UPR in immune cells promotes immunoinhibitory effects. Exposure of immune cells to factors derived from tumors can induce the UPR. Tumor lipids can induce IRE1α in myeloid cells and promote immunosuppressive function. In dendritic cells, expression of IRE1α results in aberrant lipid metabolism and results in excess lipid droplet formation and impaired Ag presentation. Additionally, loss of BAT3 can promote IRE1α and alter metabolite production to favor glucocorticoids. The release of glucocorticoids into the microenvironment could suppress the effector function of T cells. Furthermore, activation of IRE1α in T cells perturbed *N*-linked glycosylation and mitochondrial respiration, resulting in decreased IFN-γ production and increased expression of exhaustion markers. PERK expression in T cells was associated with the inhibition of T-bet, the master transcription factor mediating Th1 differentiation, as well as reduced glycolysis and increased mtROS production. In macrophages, IRE1α promotes angiogenesis and tolerogenic phenotypes while suppressing phagocytosis. Although the tumor-derived factors that induce PERK activation are not yet understood, its activation in TAMs enhances mitochondrial function and epigenetic reprogramming, leading to increased immunosuppressive function. In myeloid-derived suppressor cells, PERK promotes redox homeostasis and mitochondrial fitness, resulting in enhanced immunosuppressive function.

External factors within the TME such as nutrient scarcity, hypoxia and acidosis, metabolites, and reactive oxygen species (ROS) can perturb ER homeostasis in immune cells, leading to malfunctional innate and adaptive immunity (Fig. 2).

To begin, macrophages are a highly heterogeneous subset of innate immune cells whose primary functions include sensing and sampling the surrounding microenvironment. As cues within the microenvironment change, macrophages undergo a phenotypic shift that can mediate inflammatory diseases (65, 66). It is well documented that tumor-associated macrophages (TAMs) represent the most abundant immune cell within many types of tumors and display protumoral features. Importantly, it has been suggested that the activation of UPR signaling is critical and can facilitate this phenotypic shift. It has been demonstrated that tumor-conditioned media can induce the levels of XBP1s, eliciting the immunosuppressive function of macrophages in vitro. This increase in XBP1s was found to promote the expression of angiogenic factors within macrophages, likely through phosphorylation of STAT3 and STAT6 (46, 67–69). Moreover, as described above, IRE1α-XBP1 is also thought to mediate angiogenic factors in the tumor cells themselves, indicating that the IRE1α/XBP1 axis may be crucial for mediating angiogenesis in both cancer cells and TAMs. Additionally, the induction of XBP1s in macrophages was found to regulate the expression of ligands for “don’t eat me” signals. In human macrophages derived from patients with colorectal cancer, XBP1s induction correlated with the expression of THBS1 and signal regulatory protein α (SIRPα), which bind to CD47 and suppress phagocytosis, suggesting that XBP1s may play a role in preventing tumor cell clearance (46).

Previous studies have demonstrated that tumor-derived factors can potently induce the UPR signaling in immune cells. For example, when macrophages were exposed to conditioned media from thapsigargin-treated prostate cancer cells, melanoma cells, or Lewis lung carcinoma, they exhibited an ER stress response in vitro (70). Notably, these macrophages showed an inflammatory phenotype, which could potentially account for the coexistence of both inflammatory and anti-inflammatory macrophages in the tumor microenvironment. Additionally, recent evidence suggests that tumor-derived lipids can potently induce the activation of the UPR in immune cells. It has been reported that in ovarian cancer, DCs exhibit high levels of intracellular ROS and lipid peroxidation, which significantly induce IRE1α-XBP1, resulting in aberrant DC immunological function (71). This elevated XBP1 signaling was suggested to mediate a lipogenic program leading to an accumulation of lipids and excess formation of lipid droplets that impair antitumor immunity (71–73). Excess lipids within DCs were found to limit Ag presentation to T cells, potentially due to IRE1α-driven degradation of MHC class I (71, 74). However, with loss of IRE1α or XBP1 in DCs, the aberrant lipogenesis observed was reversed and Ag presentation was restored (71–74).

Tumor-derived cholesterol has also been of increased interest for its role in modulating the immunosuppressive phenotype of myeloid-derived suppressor cells (MDSCs). MDSCs are a group of immature myeloid cells that are highly immunosuppressive (75). Importantly, this immunosuppressive function can be induced by XBP1. It has been documented that XBP1 can regulate cholesterol biosynthesis in tumor cells, which can then be released into the tumor microenvironment to activate MDSCs (76). Perturbation of the IRE1α/XBP1 axis or cholesterol biosynthesis in the tumor could prevent MDSCs from acquiring immunosuppressive function in murine melanoma and colon cancer models. Moreover, XBP1 splicing was not observed to occur in MDSCs after exposure to cholesterol, suggesting that cholesterol-mediated MDSC phenotypes may be independent of MDSC-intrinsic XBP1s.

ER stress in macrophages has also been reported to be caused by the uptake of oxidized low-density lipoproteins via CD36 (77). In melanoma, β-glucosylceramides were found to ligate the receptor Mincle on macrophages, altering the lipid composition of the ER and inducing IRE1α activation (78). IRE1α was then able to complex with STAT3 to induce its phosphorylation and regulation of immunosuppressive genes. Interestingly, this signaling axis was not able to suppress the expression of arginase 1 (Arg1), a common immunosuppressive marker found in TAMs from many cancers, suggesting that other factors or other arms of the UPR may be necessary for TAMs to undergo a completely immunosuppressive program (78). Indeed, another study demonstrated a relationship between IRE1α-XBP1s and PD-L1 expression that may be mediated by RIDD (79).

In addition to IRE1α-XBP1, the TME was found to induce PERK expression in tumor macrophages and promote immunosuppressive activity. A recent study uncovered that PERK activation was necessary to mediate the biosynthesis of serine via ATF4 (34). When PERK signaling was perturbed, intracellular levels of serine significantly decreased, resulting in major dysregulation of mitochondrial respiration and epigenetic function, including gene expression of Arg1 (34). Moreover, this study highlighted a role for ER stress signaling as a major cellular hub or bottleneck of activity whereby targeting ER stress pathways may effectively shut down numerous processes required for cell survival in the TME.

In addition to PERK’s role in macrophages, PERK was found to play an important role in the immunosuppressive function of MDSCs in the TME. In these cells, PERK was found to regulate NRF2 antioxidant signaling to maintain redox homeostasis; however, when PERK was lost, this impaired mitochondrial ROS (mtROS) homeostasis, increasing mitochondrial damage, and induced type I IFN production through STING. This was able to increase the activity of antitumor CD8^+^ T cells and reduce tumor burden in several murine solid tumor models (80). Another important aspect of myeloid polarization toward protumoral phenotypes is the enhanced production of immunosuppressive metabolites to perpetuate a stressful and suppressive tumor microenvironment. In DCs, loss of BAT3, an adaptor protein that binds to TIM-3 on T cells, induced an overactivation of the UPR (i.e., Xbp1s, Ddit3, Atf4, Grp78, Gadd34) that shifted DC metabolism toward glucocorticoid production (81). These glucocorticoids were able to be released into the TME where they could strongly suppress T cells in a paracrine manner.

It is apparent that activation of UPR signaling in myeloid cells induces a protumoral shift in cellular function. However, induction of ER stress signaling in lymphocytes such as T cells may instead result in immunological dysfunction and ultimately exhaustion. Tumor-infiltrating lymphocytes were demonstrated to display an increase in XBP1 activity, which was markedly associated with dysregulated *N*-linked glycosylation, perturbed mitochondrial respiration, and decreased IFN-γ production in patients with ovarian cancer (82–84). In the TME, tumor-infiltrating lymphocytes are unable to adequately uptake essential nutrients, and they exhibit a noticeable deficiency in glycolysis and glutaminolysis, which results in dysregulation of mitochondrial activity and antitumor capacity (82).

In addition to nutrient deprivation, excess lipids in the TME have been suggested to induce ER stress, thereby affecting T cell antitumor immunity (85, 86). It has been reported that elevated levels of cholesterol in melanoma could induce ER stress in cytotoxic CD8 T cells. This tumor-secreted cholesterol resulted in T cell exhaustion in which activation of IRE1α-XBP1 induced the expression of PD-1, TIM-3, and LAG-3 (87). Ablation of XBP1 or cholesterol metabolism abrogated the exhausted phenotype, restoring T cell antitumor immunity (82, 87).

In multiple murine tumor models, dysregulated T cell responses have been linked to PERK signaling, which appears to involve the repression of T-bet, the master transcriptional regulator for Th1 differentiation. Specifically, high expression of the CHOP protein has been implicated in this process, potentially shedding light on the underlying mechanisms of T cell dysregulation in cancers (88). Moreover, chronic PERK-CHOP signaling was found to induce mtROS and negatively regulate glycolytic metabolism (88, 89). This seems to suggest that IRE1α may serve to negatively regulate mechanisms by which T cells acquire nutrients such as glucose and glutamine, whereas PERK negatively regulates the pathways that would use those nutrients in the TME. Taken together, this combination would significantly limit the ability for T cells to differentiate into proinflammatory phenotypes, let alone exert effector function in cancer.

### What is next in the UPR and cancer immunotherapy?

The recent interest in ER stress and the UPR has unveiled new facets of cancer biology and tumor immunology and highlighted these interactions as important aspects of tumorigenesis. It can now be appreciated that ER stress induction in tumor cells provides beneficial functions that protect the tumor against hypoxia and apoptosis while at the same time mediating angiogenesis and metastasis. Moreover, the UPR can induce the production of immunosuppressive mediators that can be released into the TME and exert potent effects on infiltrating immune cells. Myeloid cells, in particular, are extremely sensitive to changes to the microenvironment, and the influx of tumor-derived lipids, among other unidentified factors, imparts a phenotypic shift toward protumoral activity, thus adding further benefit to the malignant cells. These immunosuppressive myeloid cells can then exert their own inhibitory function upon cytotoxic cells such as T cells and affect their antitumor behaviors.

Importantly, each arm of the UPR seems to perform its own primary function in relationship to the others. Although we would be remiss to assume that these functions are universal to all tumor types or pathologies, we can use this information as a means to explore what other aspects of tumorigenesis each arm controls. Notably, ATF6 is the least studied pathway; however, it has been reported that ATF6 may be crucial for the acquisition of immunoinhibitory activity by polymorphonuclear MDSCs in the TME. Deletion of ATF6 in polymorphonuclear MDSCs promoted Ag-specific responses and delayed tumor growth in animals (90). Although some studies suggest that ATF6 may play roles in protecting against apoptosis, its roles still need to be clarified in the TME. To this end, it still remains to be seen how each signaling cascade can interplay with the others to exert optimal function. We raised the question above about whether IRE1α can mediate nutrient acquisition while PERK mediates nutrient usage. Should this be the case, determining how each arm coordinates these varying aspects would be immensely beneficial to the field. In doing so, we may be able to identify convergence points that can be targeted to optimally suppress the UPR function. Until then, we should consider multitargeted approaches that inhibit the UPR in its entirety and determine whether this can then synergize with therapies enhancing T cell responses in cancers.

Another area of interest lies with BiP, the ER chaperone that inhibits the three arms during homeostasis. It is not clear what the inciting factor is that dissociates BiP from the sensory proteins of the UPR during tumorigenesis. It would also be interesting to consider whether UPR activation can be induced independently of BiP dissociation. Another interesting point to consider is that BiP has been observed at the cell surface of tumor cells, an area where it is not canonically found (11, 91). How BiP travels to the cell surface is unclear; thus, it would be interesting to investigate the role of BiP in this location and whether it is able to communicate with the ER-associated BiP to mediate ER stress responses.

One other aspect to consider is that genetic alterations/mutations can provoke ER stress to activate the UPR. It has been suggested that oncogenic BRAF mutation persistently induces ER stress to sustain cell survival in melanoma (92). It would therefore be intriguing to explore whether oncogenic transformation can influence the activity of the UPR, and whether this can result in changes in secreted factors or other environmental cues that influence effector function in immune cells for immune evasion. Furthermore, intriguingly, it has been suggested that tumor cell aneuploidy may be linked with the activation of IRE1α and PERK signaling of the UPR (93). It has been observed that conditioned media from aneuploid cells, when added to cultures of murine macrophages or T cells from healthy individuals, may boost the transcription of Arg1 or reduce the expression of IFN-γ and granzyme B, respectively (93). This finding sheds light on the potential influence of aneuploidy-driven UPR activation on immune cells in the TME.

## Conclusions

The UPR is recognized as a significant component of numerous pathologies. Ongoing research aims to explore how we can manipulate or disrupt these signaling cascades to optimize cancer immunotherapy. In this mini-review, we have aimed to illustrate how the UPR signaling can be harnessed by tumor cells to affect the microenvironment and immune cells that infiltrate the tumor. Gaining a deeper understanding of these pathways and their interactions may reveal innovative strategies for combating cancers and improving patient outcomes.
